# Impact of briefly-assessed depression on secondary prevention outcomes after acute coronary syndrome: a one-year longitudinal survey

**DOI:** 10.1186/1472-6963-6-9

**Published:** 2006-02-13

**Authors:** Hannah M McGee, Frank Doyle, Ronán M Conroy, Davida De La Harpe, Emer Shelley

**Affiliations:** 1 Health Services Research Centre, Department of Psychology, Royal College of Surgeons in Ireland, 123 St. Stephen's Green, Dublin 2, Ireland; 2 Department of Epidemiology and Public Health Medicine, Royal College of Surgeons in Ireland, 123 St. Stephen's Green, Dublin 2, Ireland

## Abstract

**Background:**

Patients with acute coronary syndromes (ACS) are at increased risk of further acute cardiac events. Secondary prevention aims to decrease morbidity and mortality post-ACS. Depression is related to increased risk in this population, and to poorer secondary prevention activities. However, lengthy depression assessment techniques preclude depression assessment in routine care. The present study investigated the relationship of briefly-assessed depression with secondary prevention outcomes one year post-ACS.

**Methods:**

Following ethics committee approval, hospitals recruited patients for a national survey of ACS. Consenting patients with ACS completed a brief depression scale during hospitalisation. The predictive validity of two brief scales was independently assessed, with groups combined for the overall sample. Participants then completed a one-year longitudinal follow-up postal survey of secondary prevention activities.

**Results:**

The response rate for follow-up was 86% (n = 681). Proportions taking anti-platelet (88% v 87%; p = 0.334) and lipid-lowering (83% v 84%; p = 0.437) therapies remained unchanged. Prevalence of smoking (40% v 22%; p < 0.001), and median number of cigarettes smoked (20 v 10; p < 0.001) were significantly reduced at one year. Fifty-six per cent of patients reported attending cardiac rehabilitation programmes. Of those aged <65 years at baseline, 54% had returned to work at one year. A majority (56%) reported feeling physically better. Prevalence of depression was unchanged in those who completed a depression scale at both time points (15% v 17%; p = 0.434). Baseline depression did not predict taking anti-platelet, blood pressure or cholesterol medications (all p > 0.05), but did predict continuation of smoking (OR = 2.3, 95% CI 1.3–4.0, p = 0.003), a higher (above median) number of general practitioner visits (OR = 2.1, 95% CI 1.3–3.4, p = 0.005), failure to return to work (OR = 0.4, 95% CI 0.2–0.8, p = 0.015), and not feeling better (OR = 0.6, 95% CI 0.3–1.0, p = 0.05) at one year.

**Conclusion:**

Rapid depression assessment can be used to help identify patients with ACS at risk of a range of poorer secondary prevention outcomes. The results provide support for the routine screening of depression in acute settings. Strategies to increase rates of smoking cessation, return to work, general well-being and decrease health service use by depressed patients may need to incorporate some element of treatment for depression.

## Background

Patients with acute coronary syndrome (ACS; unstable angina or myocardial infarction) are at increased risk of cardiovascular morbidity and mortality, and therefore secondary prevention is an important consideration for health providers [1-3]. Reductions in subsequent morbidity and mortality have been established through the use of anti-platelet, anti-hypertensive and lipid-lowering medications; participation in exercise, dietary and cardiac rehabilitation programmes; and through smoking cessation [1-3]. However, there is ample evidence that recommended secondary prevention targets are not currently being met [4-7]. Furthermore, patient benefit may be less than assured since patient adherence to prescribed medications may be substantially poorer than prescription rates recorded in medical records [8,9]. Therefore it is important to monitor the current self-reported secondary prevention behaviours of patients with ACS.

Patients with ACS who also have depression are at greater risk of further cardiovascular events than those without depression [10-12]. One reason for this may be that depressed patients have poorer secondary prevention profiles [13]. Depression is known to predict outcomes in patients with ACS, including mortality, health service use and secondary prevention activities such as smoking cessation and medication adherence [10-15]. Depression is also related to other psychosocial outcomes such as returning to work after cardiovascular disease, and is associated with failing to increase leisure exercise [16-19]. The prevalence of depression remains steady for up to 5 years following hospitalisation for ACS [20]. Depressed patients rate their quality of life as being lower after cardiac illness [21]. Although the treatment of depression post-ACS has not been shown to reduce morbidity and mortality, it does improve quality of life [22]. Therefore, the identification of depression in ACS patients is a significant concern for the health services, as it increases burden for services, and negatively affects health outcomes and quality of life.

It is important to profile surviving patients for effective planning and evaluation of ongoing care. The continuing prevalence of depressive symptoms in this population is unknown, and knowledge of any possible consequence of depression for secondary prevention outcomes is essential for health professionals. Most research has used clinical interviews or lengthy self-completion questionnaires to assess depression while patients are still in coronary care settings [10,11,22]. These complex methods mitigate against routine depression measurement in the majority of acute settings, and make health services research time-consuming for both patients and researchers. To become a standard aspect of coronary care, simple, rapid depression assessment is required. The present study surveyed a national cohort of Irish patients one year following hospital admission for confirmed ACS [23,24], and assessed the impact of briefly-assessed depression on their secondary prevention profile. Hospitals were randomly assigned one of two brief scales – each patient completed only one scale to minimise research burden in this acute hospital period. Depressed cases from either scale were combined for analysis. The results were also analysed for each scale separately and the findings compared.

## Methods

### Participants and procedure

**Figure 1:**
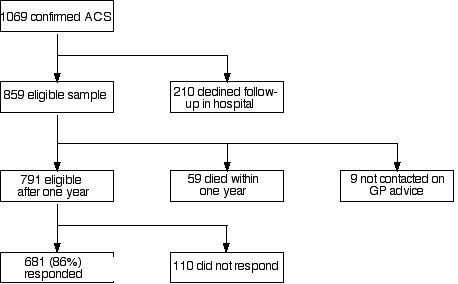
Flowchart of patient participation.

The baseline methodology has been described previously [23,24]. Briefly, after receiving ethical approval [25], all Irish centres admitting ACS patients to intensive/coronary care were invited and agreed to participate in a survey focusing on time-to-treatment and reperfusion (thrombolysis and/or direct infarct angioplasty) for eligible patients. Consecutive suspected ACS patients were recruited by staff to participate in the survey, until 25 suspected acute myocardial infarction patients were recruited. Patients were asked for consent to participate in a one-year outcomes survey during index hospitalisation, and were given a depression questionnaire. Surviving patients (as confirmed by their general practitioner) were surveyed by post 12 months later. They received a questionnaire assessing secondary prevention activities, cardiac rehabilitation, health service use, physical health rating, return to work, and a repeat depression questionnaire. Non-responders were sent postal reminders after two and four weeks. Data are presented on confirmed ACS patients who responded to the postal questionnaire (Figure 1).

### Depression measures

Hospitals were randomly assigned either the 7-item Hospital Anxiety and Depression Scales – depression subscale (HADS-D) [26] or the 7-item Beck Depression Inventory – Fast Scale (BDI-FS) [27]. Both scales use a four-answer option format, and item scores range from 0 to 3, with higher scores indicating greater symptom severity. In line with previous research, HADS-D scores >7 [28], and. BDI-FS scores >3 [27] were defined as indicating depressed cases. Both scales can be completed within minutes, and have sensitivity and specificity of >0.80 [27,28]. Patients scoring above cut-off on whichever scale they completed were defined as depressed, providing the overall sample of cases of depression.

### Statistical analysis

Analysis was conducted using STATA/SE 8.2, using robust variance estimation commands to account for original clustering of patients within hospitals. Median and inter-quartile range (IQR) are reported for ordinal data, with changes between baseline and one year tested using the Wilcoxon matched-pairs signed-ranks test. Logistic regression predicted odds-ratios (OR) for event occurrence. Unadjusted ORs are presented, unless stated otherwise. Age was analysed as a continuous variable with odds ratios calculated for a 1-year age increase, other variables were analysed as dichotomous. McNemar's test was used to compare dichotomous variables between baseline and one year. Spearman's rank correlation was used to test relationships between variables which were not normally distributed. A median split was then utilised in order to elicit an OR for event occurrence. To compare the depression scales, dummy variables were created for the total sample (i.e. scoring above cut-off for HADS-D or not – all participants who completed a BDI-FS are given a score of 0 in this variable, and vice versa). Both dummy variables were incorporated into a model, and a post-hoc (Wald) test then examined whether one scale was superior to the other as a predictor for event occurrence.

## Results

### Responders

Of the eligible 791 patients, 681 responded to the survey questionnaire (86% response rate: 76% men, mean age 63 years, std dev = 12)(see Figure 1). Non-respondents did not differ to respondents on age, sex, history of hypertension, smoking, acute coronary syndrome, revascularisation or total cholesterol level (data not shown, all p > 0.10). However, responders were marginally more likely to have private health insurance (OR 1.5, 95% CI 0.99–2.24, p = 0.055), marginally less likely to have diabetes (OR 0.61, 95% CI 0.37–1.01, p = 0.054), and marginally less likely to be depressed at baseline (OR 0.56, 95% CI 0.31–1.03, p = 0.064).

### Paired comparisons of baseline and one-year characteristics

The baseline and one year self-report outcomes are displayed in Table 1. At baseline and one year, similar proportions of patients were taking aspirin and lipid-lowering medications, while significantly fewer reported taking medication for blood pressure. Both proportion of smokers, and number of cigarettes smoked, were significantly reduced at one year, and the prevalence of depression had been maintained. The proportions of those aged <65 years in full-time employment had decreased. Of the current sample, 36% had a previous history of ACS.

### Medications

Baseline depression was not predictive of self-reported use of aspirin (OR = 0.5, 95% CI 0.3–1.2, p = 0.110), blood pressure (OR = 0.9, 95% CI 0.5–1.7, p = 0.731) or cholesterol medications at one year (OR = 0.6, 95% CI 0.3–1.2, p = 0.164). At one year, proportions of patients taking medications for depression, anxiety and sleep were 10%, 8% and 15% respectively. Patient reports showed that those depressed at baseline were more likely to be taking anti-depressant (OR = 4.1, 95% CI 2.3–7.1, p < 0.001), anxiolytic (OR = 4.1, 95% CI 1.9–8.9, p = 0.001) or sleeping (OR = 3.2, 95% CI 1.8–5.7, p < 0.001) medications at one year. Both the baseline and HADS-D and BDI-FS scales predicted taking these medications at one year (data not shown, both scales p < 0.01 for each variable).

### Health advice, smoking and service use

The majority of patients reported receiving dietary (77%) and exercise advice (81%), with no influence of baseline depression (data not shown, p > 0.05).

Patients who were depressed at baseline were no more likely to be current smokers at index admission (OR = 1.5, 95% CI 0.96–2.3, p = 0.073), but were significantly more likely to continue to smoke after hospitalisation (OR = 2.3, 95% CI 1.3–4.0, p = 0.003), even when controlling for a prior history of ACS (OR = 2.4, 95%CI 1.4–4.1, p = 0.003). However, the HADS-D did not predict continuation of smoking at one year (OR = 1.6, 95% CI 0.6–4.3, p = 0.377), but the BDI-FS did (OR = 2.6, 95% CI 1.4–5.2, p = 0.007).

Patients reported attending a general practitioner a median of 5 (IQR 3–10) times in the year following index admission, with 3% recording no visits to their general practitioner. Spearman's correlation showed a significant positive relationship between baseline depression and number of general practitioner visits (rho = 0.132, p = 0.006). A median split was made on the number of general practitioner consultations, with those attending 6 or more times defined as 'frequent users'. Logistic regression determined that those depressed as baseline were more likely to be frequent users (OR = 2.1, 95% CI 1.3–3.4, p = 0.005), even when controlling for age and sex (OR = 2.0, 95% CI 1.2–3.2, p = 0.008). Depression also predicted frequent users when controlling for prior history of ACS (OR = 2.1, 95% CI 1.3–3.4, p = 0.005). This finding was repeated for those who completed the HADS-D (OR = 2.7, 95% CI 1.2–5.9, p = 0.017), but the association was weaker and not statistically significant for the BDI-FS (OR = 1.7, 95% CI 0.8–3.3, p = 0.131).

Fifty-six per cent of patients reported attending an outpatient cardiac rehabilitation programme. Depression did not predict cardiac rehabilitation attendance (OR = 0.6, 95% CI 0.4–1.1, p = 0.120), and remained non-significant when controlling for prior ACS (OR = 0.7, 95% CI 0.4–1.2, p = 0.148). The HADS-D did not significantly predict cardiac rehabilitation attendance (OR = 0.7, 95% CI 0.3–1.9, p = 0.467), but the BDI-FS did (OR = 0.6, 95% CI 0.3–1.0, p = 0.039), with depressed BDI-FS cases less likely to attend.

### Psychosocial outcomes

Of those patients who were pre-retirement age (aged <65 on index admission, n = 363), 195 (54%) stated that they had returned to work at one year. For these patients, the median duration to return to work was 8 (IQR 4–16) weeks. Depressed patients were less likely to return to work (OR = 0.4, 95% CI 0.2–0.8, p = 0.015), even when controlling for prior ACS (OR = 0.5, 95% CI 0.2–0.9, p = 0.025), or when controlling for age and sex (OR = 0.4, 95% CI 0.2–0.7, p = 0.004). Depressed HADS-D cases were less likely to return to work (OR = 0.2, 95% CI 0.06–0.6, p = 0.007), but this did not occur for BDI-FS depressed cases (OR = 0.7, 95% CI 0.3–1.7, p = 0.444).

Patients were asked to rate their own health since the index hospitalisation. Patients reported feeling better in the majority of cases (56%), and feeling the same in almost a third of cases (31%). 'Don't know' was reported by 3%, while 10% of patients reported feeling worse at one year. This variable was dichotomised into 'better or not' to determine predictors of feeling better. Patients who were depressed at baseline were less likely to feel better one year later (OR = 0.6, 95% CI 0.3–1.0, p = 0.05), but this effect became marginal when controlling for prior ACS (OR = 0.6, 95% CI 0.4–1.04, p = 0.068), or when controlling for age and sex (OR = 0.6, 95% CI 0.4–1.04, p = 0.071). While the effect of depression on subjective health was similar in the subgroups receiving the HADS-D (OR = 0.5, 95% CI 0.2–1.2, p = 0.114) and BDI-FS (OR = 0.6, 95% CI 0.3–1.2, p = 0.123), the associations were not significant.

Patients reported living with a median of 1 (IQR 1–2) other person, with 23% of patients living alone. Depression at baseline (OR = 0.8, 95% CI 0.4–1.7, p = 0.555) or one-year depression (OR = 0.7, 95% CI 0.4–1.3, p = 0.251) was not related to living alone.

### Depression status

Baseline depression and response rates for the overall sample are reported elsewhere [29]. The response rate for completing a depression scale in the follow-up sample was 88% (598/681). Patients were more likely to complete a HADS-D (94%) than a BDI-FS (82%) (OR = 3.4, 95% CI 2.0–5.7, p < 0.001). Patients completed either a HADS-D at both baseline and follow-up (n = 254), or a BDI-FS at both baseline and follow-up (n = 193).

**Figure 2:**
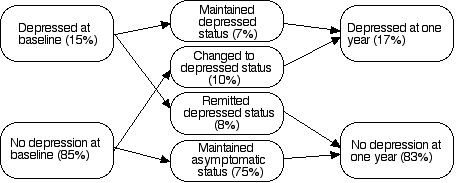
Change in depressive status between baseline and one year.

Median score for the HADS-D at baseline was 4, and this was not significantly different to median score of 3 at one-year follow-up (z = 0.259, p = 0.796). For the BDI-FS, median baseline score was 1, and one-year median score was 0 (z = 0.141, p = 0.888). However, this disguises a substantial switch in depression status of a number of patients (Figure 2). Eight per cent of the sample who were depressed at baseline were categorised as not depressed at one year. Conversely, 10% of the sample who were not depressed at baseline were classified as depressed at one year. This change in depression status was seen for both instrument subgroups. For the HADS-D, 15% of this sub-sample not depressed at baseline became depressed at one year, whereas 7% of the BDI-FS subgroup who were not depressed at baseline became depressed at one year. Seven per cent of patients were depressed at both baseline and follow-up. Those depressed at baseline were more likely to be depressed at one year (OR = 6.6, 95% CI 3.7–11.8, p < 0.001). Controlling for baseline depression, age (OR = 0.97 for 1 year increase, 95% CI 0.94–0.996, p = 0.025) was a significant predictor of one-year depression, but sex (OR = 1.1, 95% CI 0.6–2.2, p = 0.734), and prior ACS (OR = 1.5, 95% CI 0.8–2.7, p = 0.186) were not. Participants with a discharge diagnosis of confirmed acute myocardial infarction (vs. unstable angina) were less likely to be depressed at one year (OR = 0.5, 95% CI 0.3–0.9, p = 0.014), when controlling for baseline depression.

### Post-hoc scale comparison

Although both scales predicted different outcomes, the ORs were sometimes very similar. Therefore, in order to establish whether scales were performing differently to each other (as opposed to performing differently to chance alone), Wald post-hoc tests were conducted. The scales did not differ from each other on predicting taking anti-depressant, anxiolytic or sleeping medications (p > 0.05 for each), continuation of smoking (p = 0.287), frequent visits to general practitioners (p = 0.188), cardiac rehabilitation attendance (p = 0.516), feeling better (p = 0.457), or being depressed (p = 0.353) at one year. However, the HADS-D was significantly different to the BDI-FS when predicting return to work (p = 0.026).

## Discussion

This survey outlines the one-year secondary prevention profile of ACS patients in Ireland, and the impact of briefly-assessed depression on this profile. Baseline depression, as assessed by short-form depression scales, predicted secondary prevention and psychosocial outcomes. Results are discussed in terms of secondary prevention, psychosocial outcomes, the impact of depression, comparison of depression assessment scales, implications for research and practice, and study limitations.

### Secondary prevention profile and psychosocial outcomes

The proportions of patients taking anti-platelet (87%) and lipid-lowering (84%) therapy at one year compares favourably to previous research [4-7]. A reported significant decrease in 'blood pressure' medications was puzzling – it may indicate that patients do not classify the medications they are taking as being antihypertensive medications; they may be otherwise classified e.g. 'water tablets'. Since proportions taking both aspirin and lipid-lowering medications did not change, there is little evidence to suggest that an overall reduction in adherence to secondary prevention medication was seen in the sample.

The prevalence of smoking (22%) one year post-ACS is in line with other research [6,7]. Cardiac rehabilitation attendance was reported by 56% of the sample. Given the propensity for cardiac rehabilitation to reduce clinical morbidity and mortality [3], this finding is a cause for concern. The majority of eligible patients reported returning to work after a median of eight weeks, while over half (56%) of patients reported feeling better one year after the ACS event. Overall, the secondary prevention profile of the sample was comparable with other research [4-7], and these results should therefore have good generalisability.

### Impact of depression

Previous research found that depressed patients reported taking cardiovascular medications less often than those without depression [13]. In contrast, depressed patients in the current study were not less likely to report taking cardiovascular medications. Although depressed patients in the current study reported still taking these medications at one year, it is unknown if these patients take them less often than recommended. Depressed patients were more likely to be taking antidepressant, anxiolytic and sleep medications at one year – this provides a form of validation for the short-form depression scales.

Baseline depression predicted continuation of smoking, as has been found in other research [15]. Smoking cessation strategies may thus need to incorporate some aspect of depression measurement and treatment in those patients who find it difficult to stop smoking.

An increased number of general practitioner visits were made by depressed patients, and depressed patients were also less likely to return to work. These findings remained even when controlling for age and sex, and are consistent with previous research [16,19]. These results represent further costs of depression for patients, employers and health providers, and support other research which has found that depressed patients are an increased burden on healthcare resources both in terms of usage and cost [14,16,19].

Baseline depressed cases were less likely to feel better, but this relationship became marginal when controlling for gender (supporting a hypothesis of worse psychosocial outcomes for women post-ACS – data not shown). Overall, these results imply that not only do depressed patients have worse outcomes in terms of mortality (hazard ratio = 2.8 for one-year mortality [29]) and health service use, but that the surviving depressed patients also perceive their general health outcomes to be worse. These effects were maintained when controlling for a proxy of severity of ACS (i.e. length of hospital stay – data not shown), showing the robust nature of these findings, and highlighting the importance of treating depression in this population.

Importantly, a prior history of ACS had little impact on secondary prevention outcomes in the current sample. This indicates that the depression scales were robust in predicting outcomes, regardless of previous cardiovascular history.

Seven per cent of patients were depressed at both baseline and follow-up. It is not known from the data whether these patients fluctuated between depression or no depression, or whether they were consistently depressed throughout the year post-event. Fluctuations in depressive status are not uncommon in research with hospital patients [20,30-32]. It was also possible that the patients in the current sample were depressed prior to the acute episode. Previous research has shown that those with more severe depression, and a history of depression, have worse outcomes [33,34]. Also of note in the current sample was that younger patients were more likely to be depressed at one year, but women were not. That younger patients were more likely to be depressed supports previous findings [22,32], but the absence of a relationship between depression and sex contradicts previous research [16]. Sex was not related to baseline depression (data not shown). It may be that in the present survey women did not find the acute cardiac event any more stressful than men, therefore this relationship remained non-significant at one year.

Those discharged with confirmed myocardial infarction (vs. unstable angina) were less likely to be depressed at one year. This is surprising, given that myocardial infarction would be considered a more stressful event. However, previous research has found a higher prevalence of depression in personss with unstable angina than in those with myocardial infarction [10,11]. It may be that frequent episodes of recurrent angina contribute to higher rates of stress and subsequent depression. An alternative hypothesis is that depressed patients have more chest pain as part of the somatisation of their depression.

A number of mechanisms have been postulated over time to explain the relationship between cardiovascular disease and depression [35], but coverage is beyond the scope of this paper. However, more recently some researchers have postulated that depression may simply be a marker of disease severity [36]. The finding in the present study that 'depressed' patients were more frequent visitors to general practitioners may provide some support for this hypothesis. Alternatively, it may be that depressed patients have other ailments which require more frequent visits or that depression *per se *induces professional help-seeking behaviour. The data in the present study cannot clarify this point further.

Further research should concentrate on documenting the natural history of depression after an acute event, to give an accurate picture of the progress of depressive symptoms over the course of one year, and provide data on how fluctuating depressive symptoms impact on morbidity, mortality, service use and psychosocial outcomes.

### Scale comparison

Two depression scales were independently assessed in this survey. The baseline HADS-D predicted return to work and increased general practitioner visits. The BDI-FS predicted continuation of smoking at one year and cardiac rehabilitation attendance (although combined depression scores did not predict cardiac rehabilitation attendance). Neither scale independently predicted perceived health ratings at one year. When the data was combined, having depression at baseline predicted not feeling better at one year, indicating a small effect size which neither scale was sensitive enough to detect with a split sample. Post-hoc comparisons of the scales showed no significant differences between them, except when predicting return to work, where the HADS-D was superior. Since the HADS-D predicted one-year mortality [29], it could be that the more predictive or stable HADS-D depressed cases were thus eliminated from the current analysis. The BDI-FS did not predict mortality, and this could have left the more stable or predictive depressed cases to be included in the BDI-FS sub-sample. Response rates at one year showed that patients were more likely to complete a HADS-D than a BDI-FS, possibly indicating that questions on the BDI-FS scale were considered more intrusive by patients. Considering these results, we recommend that the HADS-D be incorporated into everyday coronary care practice, as it can predict useful psychosocial and health service outcomes in ACS patients and is acceptable to patients.

### Implications for research and practice

The results of the present survey show the importance and clinical utility of measuring depression with short-form depression scales. Baseline depression was predictive of outcomes in a group of patients with a relatively good secondary prevention profile, and routine assessment would identify those at increased risk of poorer outcomes. Overall, these findings indicate that short-form depression questionnaires are an acceptable substitute for clinical interviews in a setting where depression would not be routinely assessed, and would provide evidence that a patient may require more thorough assessment. This is an important finding for health services research, as it shows that complex methodologies can be replicated in a more simplified manner, with less pressure on patients, service providers and researchers. Obviously, clinical interviews and longer questionnaires provide superior quality data, but these options are not feasible in all acute settings for either the health provider or the patients.

Identification of depressed patients is advisable for both service providers and patients. The prevalence of depression and the poorer outcomes seen in this group provide support for the treatment of depression to enhance patients' quality of life, and to reduce the costs to service providers through reducing the negative outcomes associated with depression. The use of brief scales may be a first step towards tackling this problem in patients with ACS.

### Study limitations

This study has several limitations. The power of the study was restricted by the eventual sample size at follow-up, and by the reduced numbers who completed a depression scale at baseline. This was evidenced by wide confidence intervals when predicting outcomes, and results must be interpreted with caution. The effects of selection bias must be considered given that respondents were more likely to have private insurance and less likely to have diabetes or be depressed at baseline. It may be that the full impact of depression is not being assessed in the present study, due to depressed patients being less likely to respond at one year. Depression may therefore have an even greater impact than that shown in this study.

There is also the potential for unmeasured confounding in the results. For example, it may be that the persons indicated as depressed may have had a more sedentary lifestyle. This may then have influenced outcomes such as cardiac rehabilitation attendance, return to work, or not feeling better. The interaction of depression and other outcomes may be more complex than these results suggest.

Since the present study was based in one country, results may not be generalisable to other countries for two reasons. Firstly, the secondary prevention profile compares favourably to that in other European countries [4-7]. Secondly, depressive symptoms may be experienced differently in different cultures/countries. However, at least two large studies [37,38] have shown that the impact of depression on physical health is similar across populations and countries. Thus there is some evidence supportive of cross-cultural generalisability.

## Conclusion

Rapid depression assessment can be used in health services research to help identify those at risk of a range of poorer secondary prevention outcomes. The results provide support for the routine screening of depression in acute cardiac patients. Strategies to increase smoking cessation, return to work, general well-being and decrease health service use by depressed patients may need to incorporate some element of depression treatment.

## Competing interests

The author(s) declare that they have no competing interests.

## Authors' contributions

HM, FD drafted the manuscript. FD carried out the survey and analysed the data. RC participated in the study design and coordination, and provided statistical input on data analysis and interpretation. HM, DD & ES conceived of the study and participated in its design and coordination. All authors provided critical input on manuscript drafts, and approved the final version.

## Pre-publication history

The pre-publication history for this paper can be accessed here:



## Figures and Tables

**Table 1:** Profile of cardiovascular medications, smoking and psychosocial status at baseline and one year post-admission

	**Baseline**	**One year**	**OR**	**95% CI**	**p-value**
**Cardiovascular Medications**					
Aspirin	88%	87%	1.2	0.8–1.8	0.334
Antihypertensive≪/Blood Pressure	94%	72%	6.8	4.5–10.7	<0.001***
Statins/Cholesterol	83%	84%	0.9	0.6–1.2	0.437
**Smoking status**					
Current Smoker	40%	22%	18.9	9.3–44.5	<0.001***
Median number of cigarettes smoked per day (IQR)	20 (18–30)	10 (7–20)	-	-	<0.001***
**Psychosocial status**					
Full/part-time employment^§^	64%	54%	2.7	1.6–4.6	<0.001***
Depression≪≪	15%	17%	0.8	0.5–1.3	0.434
*HADS-D*	14%	19%	0.7	0.4–1.2	0.169
*BDI-FS*	16%	15%	1.3	0.5–2.9	0.701

(*p < .05, **p < .01, ***p < .001)

≪ – includes angiotensin converting enzyme inhibitors, betablockers, calcium antagonists and diuretics

^§ ^– For those patients <65 years on index admission (n = 363)

≪≪ – For those who completed a depression scale at baseline and follow-up (n = 447)
